# Application of endoscopic endonasal approach in skull base surgeries: summary of 1886 cases in a single center for 10 consecutive years

**DOI:** 10.1186/s41016-020-00199-w

**Published:** 2020-06-04

**Authors:** Chuzhong Li, Haibo Zhu, Xuyi Zong, Xinsheng Wang, Songbai Gui, Peng Zhao, Chunhui Liu, Jiwei Bai, Lei Cao, Yazhuo Zhang

**Affiliations:** 1 Beijing Neurosurgical Institute, Capital Medical University, Beijing, China; 2 Department of Neurosurgery, Beijing Tiantan Hospital affiliated to Capital Medical University, Beijing, China; 3 Beijing Institute for Brain Disorders Brain Tumor Center, Beijing, China; 4 China National Clinical Research Center for Neurological Diseases, No.119 South 4th Ring West Road, Fengtai District, Beijing, 100050 China

**Keywords:** Complication, Indication, Neuroendoscopy, Skull base surgery, Endonasal approach

## Abstract

**Background:**

Endonasal endoscopic skull base surgery has undergone rapid technological developments and is now widely performed, but its strengths and weaknesses deserve further investigation and deliberation. This study was performed to investigate the surgical indications, complications, and technical advantages and disadvantages of endonasal endoscopic skull base surgery.

**Methods:**

The clinical data of 1886 endoscopic endonasal skull base surgeries performed in our ward at Beijing Tiantan Hospital from June 2006 to June 2016 were retrospectively analyzed.

**Results:**

One thousand ninety-three (73.4%, 1490) pituitary adenomas, 54 (24.9%, 217) chordomas, 28 (80.0%, 35) craniopharyngiomas, and 15 (83.3%, 18) meningiomas underwent total resection. Two patients died postoperatively, both having pituitary adenomas. Other postoperative complications included olfactory disorders (*n* = 226, 11.9%), postoperative cerebrospinal fluid leakage (*n* = 78, 4.1%), hypopituitarism (*n* = 74, 3.9%), diabetes insipidus (*n* = 64, 3.4%), intracranial infection (*n* = 36, 1.9%), epistaxis (*n* = 24, 1.3%), vascular injury (*n* = 8, 0.4%), optic nerve injury (*n* = 8, 0.4%), and oculomotor movement impairment (*n* = 4, 0.2%). In total, 1517 (80.4%) patients were followed up for 6 to 126 months (average, 42.5 months) postoperatively. A total of 196 (13.2%) pituitary adenomas and 13 (37.1%) craniopharyngiomas recurred but no meningiomas recurred. Chordomas recurred in 97 (44.7%) patients, in whom 5-year survival rate was 65%.

**Conclusion:**

Endoscopic surgery is an innovative surgical technique and the first choice for most midline extradural lesions such as chordomas, and an excellent choice for pituitary adenomas. It probably will be a good technique for many kinds of craniopharyngiomas and a common technique for most of skull base meningiomas, so the surgical indications of these cases should be chosen carefully to make good use of its respective advantages.

## Background

Endoscopic and microscopic skull base surgeries are two important aspects of skull base surgery. The rapid development of these two surgical technologies has promoted continuous improvement of skull base surgery. In endonasal endoscopic surgery, the surgeon takes advantage of the natural corridor to manage skull base lesions directly and clearly with less injury and lower complications than traditional microscopic neurosurgery. It has advantages of less brain tissue damage and quick recovery and has developed particularly rapidly during the past 20 years, in which application has gradually expanded from the sellar area to the medial area of the skull base plus most of the lateral skull base area, from epidural lesions to subdural lesions, and from pituitary adenomas to complex lesions including aneurysms. With the expansion of these indications, however, complications such as infection, cerebrospinal fluid leakage, and nasal structure and function destruction have become more concerning. How to make full use of endoscopic and microscopic techniques for such lesions has become an important issue in the field of skull base surgery. The clinical data of 1886 endoscopic endonasal skull base surgeries in our single center from June 2006 to June 2016 were retrospectively analyzed, and the advantages, surgical points, and applicability of endoscopic endonasal surgery are summarized in this paper.

## Methods

### Materials

The clinical data of 1886 endoscopic endonasal skull base surgeries performed in our ward at Beijing Tiantan Hospital from June 2006 to June 2016 were retrospectively analyzed. This study comprised 1886 patients (957 female and 929 male patients; female to male ratio, 1.03:1.00). The average disease duration was 25.2 months (range 3 days to 20 years). The indications for surgery included 1490 cases of pituitary adenomas, 217 chordomas, 35 craniopharyngiomas, 33 Rathke cysts, 18 meningiomas, 13 cerebrospinal fluid leakage, 10 osteofibrous dysplasia, 9 metastatic carcinomas, 9 osteochondromas or chondrosarcomas, 7 plasmacytomas, 6 giant cell tumors of bone, 5 cavernous hemangiomas, 4 sinonasal cancers, 3 ossifying fibromas, 3 schwannomas, and 24 other space-occupying lesions. We obtained written informed consent from each subject. The Beijing Tiantan Hospital Research Ethics Committee approved the study.

### Equipment

The neuroendoscopy system, pneumatic support arm, and rigid endoscopes (0°and 30°) were obtained from Karl Storz GmbH & Co. KG (Tuttlingen, Germany). The irrigation pump was obtained from Clarus Medical LLC (Minneapolis, MN, USA). Bipolar coagulation instruments were obtained from ERBE Elektromedizin GmbH (Tübingen, Germany). A Cavitron ultrasonic surgical aspirator was obtained from Söring GmbH (Quickborn, Germany). The navigation system was obtained from Medtronic, Inc. (Minneapolis, MN, USA). The laser system was obtained from PhotoMedex Inc. (Montgomeryville, PA, USA). The intraoperative neurophysiologic monitor was obtained from Thermo Nicolet Corporation (Madison, WI, USA). The vascular Doppler detectors were obtained from Vascular Technology Inc. (Nashua, NH, USA). The electric drills were obtained from NSK Ltd. (Ōsaki, Shinagawa-ku, Tokyo, Japan). The ultrasound system was obtained from Hitachi, Ltd. (Chiyoda-ku, Tokyo, Japan).

### Surgical method

The patients were placed in the supine position after general anesthesia with the head tilted back at 15°. After using 5% iodophor for facial disinfection and 0.05% iodophor gauze for nasal disinfection, we usually used a 0° or 30° endoscope with right nasal approach (if the lesion was mainly located on the left, we performed the left nasal approach). Whether an open contralateral nasal approach was used depended on the extent of the intraoperative exposure and the need for lesion resection. Most cases were managed using the two-person/three-hand technique, and some were managed by the two-person/four-hand or three-person/multiple-hand technique. The surgical approaches included the conventional endoscopic trans-nasal–sphenoid approach, endoscopic trans-nasal–sphenoid/ethmoid sinus–tuberculum sellae/sphenoid platform approach, endoscopic transethmoid–pterygoid process–sphenoid sinus approach, and endoscopic endonasal–sphenoid sinus–clivus approach.

After lesion resection, we used multiple techniques for skull base reconstruction based on the demands of the operation. Four skull base reconstruction techniques were used: (1) general repair: use of hemostatic fibers or gelatin sponge/artificial dura mater (absorbable/non-absorbable) if the dura mater or arachnoid was integrated and no visible cerebrospinal fluid leakage occurred during the operation; (2) multi-layer reinforcement reconstruction with free tissue grafts: use of different material in sequence of fat, artificial dura, fascia, and muscle if arachnoid gap was < 1 cm; (3) vascular pedicled nasal mucosal flap: use of pedicled nasal septum mucosal flap or pedicled middle concha mucosal flap based on multi-layer reinforcement if the arachnoid gap was > 1 cm; and (4) dural suture: use of autologous muscle fascia or artificial dura mater for patients with a higher risk of postoperative cerebrospinal fluid leakage especially in the situation that no pedicled nasal mucosal flap was available. We usually choose 6-0 or 7-0 Prolene suture and use a special needle holder or ethmoidal forceps.

## Results

### Degree of surgical resection

The degree of tumor resection was divided into four categories: total resection (no tumor residue on imaging examination), subtotal resection (> 90% resection), partial resection (70–90% resection), and partial resection (< 70% resection). Among 1490 pituitary adenomas in our center, 1093 (73.4%) underwent total resection, 161 (10.8%) underwent subtotal resection, 122 (8.2%) underwent partial resection involving 70 to 90% resection, and 114 (7.6%) underwent partial resection involving < 70% resection. Among 217 chordomas, 54 (24.9%) underwent total resection, 91 (41.9%) underwent subtotal resection, 57 (26.3%) underwent partial resection involving 70 to 90% resection, and 15 (6.9%) underwent partial resection involving < 70% resection. Among 35 craniopharyngiomas, 28 (80.0%) underwent total resection, 4 (11.4%) underwent partial resection involving 70 to 90% resection, and 3 (8.6%) underwent partial resection involving < 70% resection. Finally, among 18 meningiomas, 15 (83.3%) underwent total resection and 3 (16.7%) underwent partial resection involving 70 to 90% resection.

### Complications

Two patients died postoperatively, both were invasive pituitary adenomas. One died of intraoperative carotid artery rupture, and the other died of postoperative intracranial hematoma caused by residual tumor. Other postoperative complications included olfactory disorders (*n* = 226, 11.9%), postoperative cerebrospinal fluid leakage (*n* = 78, 4.1%), hypopituitarism (*n* = 74, 3.9%), diabetes insipidus (*n* = 64, 3.4%), intracranial infection (*n* = 36, 1.9%), epistaxis (*n* = 24, 1.3%), vascular injury (*n* = 8, 0.4%), optic nerve injury (*n* = 8, 0.4%), and oculomotor movement impairment (*n* = 4, 0.2%).

### Follow-up

In total, 1517 (80.4%) patients were followed up for 6 to 126 months (average, 42.5 months). Among these patients, 196 (13.2%) with pituitary adenomas and 13 (37.1%) with craniopharyngiomas developed recurrence; no patients with meningiomas developed recurrence. Recurrence of chordomas occurred in 97 (44.7%) patients. The 5-year survival rate was 65% with 43 deaths.

## Discussion

The skull base involves a wide variety of diseases. Because it is deeply positioned, and the surrounding anatomical relationship is complex, great operative difficulties, high incidence of surgical complications, and poor prognosis have made skull base surgery become one of the most challenging aspects of neurosurgery. Since Jankowski et al. [1] first reported endoscopic trans-sphenoidal pituitary adenoma resection in 1992, surgeons have gradually recognized the advantages of neuroendoscopy for clear exposure of skull base diseases under direct vision. Endoscopic endonasal skull base surgery has undergone rapid technological development, and its scope of application has also gradually expanded. Endoscopic endonasal skull base surgery combined with microsurgical skull surgery constitutes the foundation of modern minimally invasive skull base surgery [2–4]. The surgical quality and prognosis of skull base surgery have greatly improved through extensive application of neuroendoscopy combined with electrophysiological monitoring, neuro-navigation, ultrasound Doppler, high-speed drill, laser, ultra-suction, support arms, and other technologies and equipment. However, with continuous expansion of its applications, the disadvantages of endoscopic endonasal skull base surgery have become apparent, such as its two-dimensional view, long and narrow corridor, difficult hemostasis, high cerebrospinal fluid leakage rate, and serious damage of nasal structure [5–8]. Many reports have compared, summarized, and reflected on the advantages and disadvantages of endoscopy and microscopy in treatment of skull base lesions [9–27]. To facilitate scientific decision-making regarding different technical means to manage skull base disease and improve surgical quality, we have herein summarized our 10-year experience of endoscopic endonasal skull base surgery with an emphasis on its advantages and disadvantages.

The key points of skull base surgery are exposure, identification, and management of the lesion followed by structural reconstruction. The aim is to protect the important structure and function of the cranial base and remove the lesion to the greatest extent possible. Therefore, the principle of approach selection in skull base surgery was using the shortest distance to the lesion with the best exposure that can cause minimal tissue destruction with maximal safety lesion resection. Better clinical results can be obtained when personalized endoscopic or microscopic surgical techniques are adopted. We consider that the status and roles of neuroendoscopy varies in different skull base surgeries and it could be divided into four types based on our experience and combined the current trend in the development of skull base surgery: (1) Innovative technology: Endoscopic technology is the only choice for this disease and can solve the problems that conventional methods cannot solve. (2) Excellent technology: Endoscopy is of the highest quality choice and can overcome the shortcomings of conventional methods, making it the most effective surgical technique. (3) Good technology: Endoscopy is an alternative technique that has been developed with other minimally invasive neurosurgical techniques; it has become a useful technology for skull base diseases that can be combined with conventional methods. (4) Common technology: Endoscopy is a promising treatment choice but still requires improvement. Some surgeries can be completed with endoscopy, but whether this is the most effective method requires further research.

### Innovative technology

**Fig. 1:**
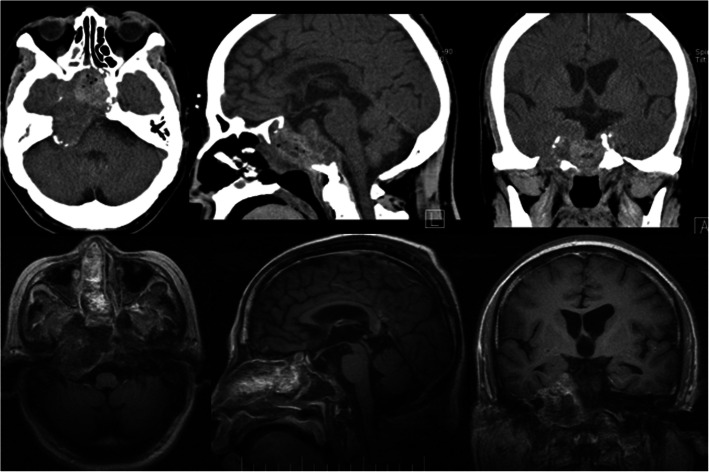
The preoperative CT (computed tomography) and postoperative MRI (magnetic resonance imaging) of a recurrent chordoma: the patient, male, 59 years old, chief complaint: trans-sphenoidal chordoma resection and radiotherapy for 6 years, right hearing loss accompanied by peripheral facial paralysis for 3 months. The tumor of sphenoid sinus, ethmoid sinus, and petrous apex region was totally resected via the endoscopic transethmoid–pterygoid process–sphenoid sinus approach and middle approach extended to petrous apex region

The indications for innovative technology mainly include skull base lesions originating from epidural diseases, especially those mainly located in the midline skull base region, such as widely growing tumors of the skull base (chordoma, nasopharyngeal angiofibroma, nasopharyngeal carcinoma, etc.), cerebrospinal fluid leakage, and orbital apex lesions. The common characteristic of these lesions is that microscopic trans-sphenoidal approach or craniotomy for tumor resection is difficult with high incidence of complications; however, the extensive exposure of skull base around the sphenoid sinus makes it relatively easier to treat lesions by endoscopic endonasal surgery. In case of chordomas which origin from extracranial, we can totally or subtotally resect the tumor through an endoscopic endonasal approach without traction or exposure of the brain tissue. The total/subtotal resection rate of chordomas in this group was 66.8%, and the incidence of complications was lower than that reported in the literature [28]. Most chordomas originated from the clivus and presented with a variety of different sizes, locations, and tumor texture consistency and blood supply. With the advantage of the wide view provided by the endoscopic endonasal approach, good exposure can be obtained for the vast majority extracranial lesions which is represented by chordoma through a process of grinding the bone of the saddle bottom, clivus, and pterygoid appropriately (Fig. 1) [29, 30]. A small amount of bleeding is encountered when managing intracranial subdural tumors because the tumor blood supply mainly originates from external carotid artery of viscerocranial region. Thus, we can carefully separate the adhesions among the intracranial tumor, including surrounding blood vessels, brain tissue, and nerves intraoperatively with little disturbance of tumor bleeding compared with craniotomy for tumor resection. Tumors with extensive invasion of the lateral skull base and brain can be resected in combination with microscopic neurosurgical technology through craniotomy approach [31, 32].

### Excellent technology

**Fig. 2:**
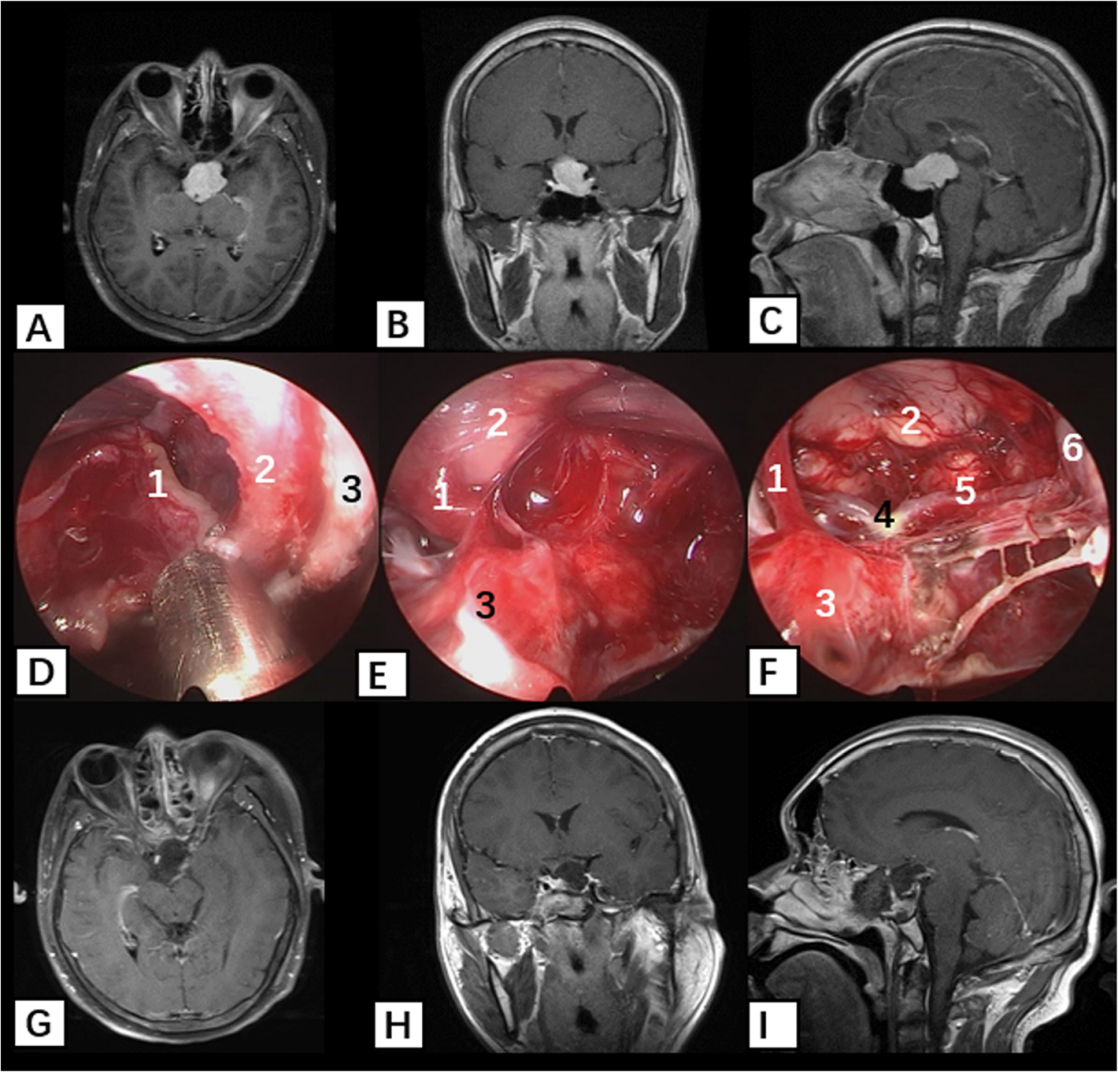
Resection of the pituitary adenomas that invade suprasellar region or clivus with endoscopic endonasal trans-sphenoidal approach. **a**–**c** Preoperative enhanced MRI T1 showed irregular abnormal signal in saddle area; **d**–**f** intraoperative findings of pituitary adenomas resection with endoscopic endonasal trans-sphenoidal approach; **d** resection of the intrasellar tumor, the left tumor boundary shows 1 tumor, 2 saddle dura, and 3 saddle bone; **e** the right tumor boundary shows 1 pituitary stalk, 2 tumor, and 3 normal pituitary; **f** after resection of the tumor, 1 pituitary stalk, 2 mamillary body, 3 normal pituitary, 4 base arterial bifurcation, 5 posterior communicating artery, and 6 left internal carotid artery; **g**–**i** postoperative enhanced MRI T1 showed total tumor resection

The representative lesion of excellent technology is the pituitary adenoma. Compared with microscopic trans-sphenoidal technology, the endoscopic endonasal approach has the advantages of wide exposure and direct vision, especially for tumors that invade cavernous sinus, suprasellar region, or clivus. In recent years, endoscopic endonasal resection of pituitary tumors has been increasing, showing strong competitiveness [33–37]. The present study included 1490 pituitary adenomas, and 1093 (73.4%) were totally resected while 196 (13.2%) recurred. These results are better than those obtained by the microscopic endonasal approach during the same period. In the present study, we resected lateral tumors that invaded the cavernous sinus or wrapped around the internal carotid artery through a trans-sphenoidal approach with lateral extension or trans-ethmoidal–pterygoid–sphenoidal approach. For tumors that extended upward and were classified as Hardy grade > III (e.g., the tumor texture was slightly tough and difficult to collapse), we resected the tumor under direct vision through an endoscopic endonasal–sphenoid–tuberculum sellae approach and achieved good clinical results (Fig. 2). Many meta-analyses have summarized the advantages and disadvantages of endoscopic trans-sphenoidal and microscopic trans-sphenoidal surgery [9, 12, 18, 19, 22, 26, 32], and most studies have concluded that no significant difference exists in the surgical resection rate or complication rate between the two procedures. Schwartz [24] summarized the studies performed in recent years and concluded that for small intrasellar tumors, both approaches appear equally effective in experienced hands. For larger tumors with extrasellar extension, the endoscopic approach offers several advantages and may improve outcomes associated with the extent of resection and postoperative complications.

### Good technology

In case of craniopharyngioma, endoscopic resection has the advantages of no traction of the brain tissue or optic nerve, early identification and protection of the pituitary stalk and hypothalamus, and separation of the tumor capsule under direct vision [27, 38–40]. Especially the advantages of close observation under endoscopy achieve a good identification and protection for the small perforated vessels which supply optic chiasm and the hypothalamus, then significantly reduce the incidence of postoperative complications. In the present study, the rate of total endoscopic resection was 80%, which was better than that achieved by craniotomy approach. The incidence of postoperative fever, electrolyte disturbances, and urinary incontinence and other hypothalamic complications was lower than that associated with microsurgery; these results are consistent with most of the literatures [15, 23]. Komotar et al. [15] summarized studies involving 3470 patients and found that the endoscopic cohort had a higher rate of gross total resection and better visual outcomes than did the open cohort. The rate of cerebrospinal fluid leakage was higher in the endoscopic (18.4%) and microscopic trans-sphenoidal (9.0%) groups than in the transcranial group, but the transcranial group had a higher rate of seizures (8.5%), which did not occur in other two groups. Another point to note is that during endoscopic surgery, we must remove the middle turbinate and posterior one third of the nasal septum, open the ethmoid and sphenoid sinus, and grind the tuberculum sellae and part of the sphenoid plate bone. The degree of nasal destruction and the risk of cerebrospinal fluid leakage are higher than in traditional surgery. Thus, microsurgery with craniotomy approach still has some advantages for patients with extensive invasion craniopharyngiomas. Because of the number of reported cases of endoscopic surgery is relatively limited, additional clinical trials are needed to prove the superiority of endoscopic technology. Especially for craniopharyngiomas mainly involving the third ventricle or exhibiting lateral expansion, craniotomy is still the first-choice treatment [23, 27].

### Common technology

The representative diseases included meningiomas at different positions of the skull base, such as the olfactory groove, tuberculum sellae, clivus, and jugular foramen. Endonasal endoscopic approach has the advantage of firstly cutting off the tumor blood supply, which makes it easier to remove the tumor. The other advantage includes that most of the invaded dura mater and skull base bone can be resected without traction on the brain tissue [41]. But the degree of nasal destruction and the risk of cerebrospinal fluid leakage are higher than those in the traditional surgery. Especially, the microsurgical resection of olfactory groove and tuberculum sellae meningiomas is quite mature, and the rate of postoperative complications is very low. We can take full advantage of endoscopic technology only by strict adherence to its indications [17, 42]. In our center, we resected all the olfactory groove meningiomas through craniotomy approach. The surgical approach for tuberculum sellae meningiomas was mainly determined according to the location of the tumor base. We resected the tumor through an endoscopic endonasal approach if the base of the tumor was located in the tuberculum sellae extending to the anterior wall of the sphenoid sinus and the tumor was mostly located below the sphenoid platform or was slightly extending into optic canals. Craniotomy was a much better choice for tumors that extended intracranially, or the tumor base was mainly located in the tuberculum sellae and sphenoid platform. The rate of total resection of skull base meningiomas was 83.3% in this study, and no patients developed recurrence. The complication rate associated with endoscopic endonasal surgery was higher than that associated with craniotomy; two patients (11.1%) with postoperative cerebrospinal fluid rhinorrhea were performed surgical repair, and three patients (16.7%) developed hyposmia. No lower clivus meningioma or jugular foramen meningioma occurred in this study, and a small number of cases are reported in the literature [43]. This technique may only be suitable for doctors with rich endoscopic experience [44, 45].

Other new techniques reported in the literatures include endonasal endoscopic approach for intracranial aneurysm clipping [46–48], brain stem lesion resection [49], and complete endoscopic transcranial skull base approach (retrosigmoid and supraorbital approach, etc.) [50–52] also belonging to the common technology. Endoscopic-assisted microsurgery is still the gold standard for treating such diseases. Additionally, the range of applications can be widened if the narrow vision, long operating distance, two-dimensional image guidance, hemostatic difficulty, and other shortcomings can be overcome through advances in technology and equipment.

## Conclusion

The endoscopic endonasal approach is becoming more widely used in skull base surgery. It is an innovative technology and the first choice for lesions in the midline area of the skull base. The endoscopic endonasal approach has the advantages of clear, wide exposure, using natural lacunas approach, and few complications. The endoscopic endonasal approach is the excellent technology for sellar lesions (pituitary adenoma as the representative), especially that it has become the best choice for the invasion pituitary adenomas which grow toward to upward and lateral. This approach can also be a good technology for subdural lesions located in the sellar area such as craniopharyngiomas and can be a common technology for other intracranial lesions such as skull base meningiomas, and the surgical indications should be strictly determined to give full play to the advantages of endoscopic techniques.

## Data Availability

Please contact author for data requests.
